# Effectiveness of the Flipped Classroom Teaching and Learning Method Among Underachievers in Physiology: Experience From a Tertiary Care Teaching Hospital

**DOI:** 10.7759/cureus.61099

**Published:** 2024-05-26

**Authors:** L. Reshma Shireesha, Yavvari Raghu Srinivas, Sharmila Nalli, Zayapragassarazan Z, Dasari Shakeela, Venkataramana Kandi

**Affiliations:** 1 Physiology, Government Medical College, Srikakulam, Srikakulam, IND; 2 Medical Education, Jawaharlal Institute of Postgraduate Medical Education & Research, Pondicherry, IND; 3 Clinical Microbiology, Prathima Institute of Medical Sciences, Karimnagar, IND

**Keywords:** physiology, medical education technologies, medical undergraduate students, teaching and learning (tl), flipped classroom

## Abstract

Introduction

Traditional classroom teaching involves a process where knowledge is disseminated to students by the teachers through a one-way process. Such a learning environment makes students passive and muted, which can be improved by alternative teaching and learning (TL) methods like the flipped classroom (FC) technique. The FC approach involves a student-inclusive TL process. FC is a student-centered approach that benefits teachers and students by emphasizing the key information during the learning process. The present study aimed to understand the efficacy of the FC TL method and evaluate students' perception of FC among underachieving first-year medical students in learning physiology.

Methods

This prospective, descriptive, and cross-sectional study was conducted on 100 underachieving first-year Bachelor of Medicine and Bachelor of Surgery (MBBS) students in the Department of Physiology at a tertiary care teaching hospital. All the participants were taught cardiovascular physiology through the FC method. The study subjects were asked to take a pretest including multiple choice questions a week before the study. The study period was four weeks, following which the students were asked to take a post-test. A questionnaire was used to understand the student's perception of FC. The responses to the questionnaire were graded based on the Likert scale.

Results

The mean scores of the post-test (19.40±4.22) were significantly (p< 0.05) greater than those of the pre-test (12.43±4.26). Regarding the perception of FC, 68% (68/100) of students agreed that the objectives, methodology, and outcomes were well-defined. Around 39% (39/100) of students strongly agreed that the study material was adequate, relevant, and easy to learn. Nearly 58% (58/100) of the students agreed that the competencies are dealt with completely in every session. Roughly 16% (16/100) of students agreed that the classroom time is sufficiently utilized for peer-based learning. Many (61%, 61/100) students agreed that sufficient time was given for learning. About 34% (34/100) of the students strongly agreed that the assessment tools were relevant. About 56% (56/100) of students agreed and 21% (21/100) strongly agreed that the FC method helped them to improve their understanding of the subject. More than half (54%, 54/100) of the students agreed and 18% (18/100) strongly agreed that the FC promoted self-directed learning. Most students (71%, 71/100) believed FC to be an interesting and satisfactory learning experience.

Conclusions

The results indicate that most students believed FC was an effective and innovative TL technique. The FC method could help underachievers improve their cognitive skills and analytical thinking and enhance exam performance.

## Introduction

Advanced medical education technologies have recently gained attention and have been applied in medical institutions globally, including India [1]. Particularly, in India these technologies were familiarized to implement competency-based medical education (CBME) which was recently introduced by the National Medical Council (NMC), the regulatory authority of medical education (ME) [2,3]. CBME was intended to produce more efficient and skillful Indian medical graduates. ME and training of students pursuing a Bachelor of Medicine and Bachelor of Surgery (MBBS) are intended to prepare them to become physicians or clinicians capable of treating and managing patients [3]. Recently, ME has been revolutionizing as evidenced by the shift from traditional didactic large-group teaching to modern student-centered approaches [4,5]. A flipped classroom (FC) is an innovative method that promotes self-directed learning (SDL), critical thinking, and peer-based learning, enabling the students to learn at their own pace. FC is an inverted classroom approach and an efficient and promising blended learning method. The FC was first introduced by Jonathan Bergmann and Aaron Sams, two high school chemistry teachers from Colorado, United States of America (USA) [6].

The traditional didactic lectures are believed to be a teacher-centered approach. In this method, the teacher assumes that the student is a passive learner and delivers the lecture. This monotonous teacher approach is intended to confer knowledge to the students, assuming they have an empty brain. However, in the FC model, the teacher takes a student-centered approach and shifts from being a sage on the stage to a guide on the side. In the FC approach, the teacher acts as a facilitator and orchestrates the context, provides the relevant resources, poses questions, and stimulates critical thinking, following which the student carries out SDL to come up with answers [7].

Traditional lectures lack tools that ensure the student’s intellectual engagement with the study material during the long didactic lectures. Disadvantages of these lectures include the quick waning of students' attention and the pace of the lectures is not adjusted to meet all learner’s needs [8]. Therefore, traditional lectures are not suited to teaching higher-order skills such as application, problem-solving, and analysis. 

This study was carried out to analyze the effectiveness of the FC method to reinforce core competencies among underachievers pursuing MBBS. We also assessed the students' perceptions of the FC in improving their learning abilities and enhancing exam performance.

## Materials and methods

This prospective, descriptive, and cross-sectional study was conducted among first-year MBBS students in the Department of Physiology, Government Medical College, Srikakulam, Andhra Pradesh, India. Institutional ethics committee approval was obtained (IEC23/GMC&GGH/ SKLM/180823/23). A total of 100 students were enrolled as participants. The study participants were categorized as underachievers based on their previous academic performances. All 100 participants were explained in detail about the study protocol and the purpose of the study and written consent was obtained.

Flipped classroom model

A pre-test was conducted one week before the beginning of the study period, using a standard questionnaire that included multiple choice questions (MCQs). The topics selected were core competencies of cardiovascular physiology. The study period was divided into four weekly sessions scheduled during the independent teaching slots for physiology. All the participants were motivated to participate in all the sessions by suggesting their usefulness in preparing for examinations. 

The study material was provided as PowerPoint presentations (PPTs), portable document format (PDF), and videos to all the participants through a WhatsApp group one week before every scheduled session. Students were instructed to watch the videos, read the study material, make mind maps, notes, and flowcharts, and write down the queries before the next FC session.

During the FC sessions, students were divided into small groups with one among them acting as a moderator. The students were encouraged to participate in small group discussions, ask questions, draw diagrams, solve MCQs, clarify misconceptions, understand the concept, and clarify doubts. Students engaged actively with the facilitator, moderator, and with each other.

**Figure 1:**
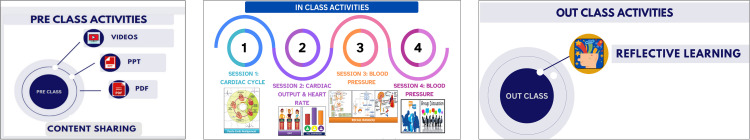
Process depicting the flipped classroom Image credit: The authors

During the first FC session, the in-class activity was designed with a team-based contest, where the students rearranged the shuffled cards with images depicting the sequence of various phases of the cardiac cycle. In the second session, the recall of the competencies like cardiac output and heart rate were analyzed, which were learned through quizzes. During the third session of the FC, the theoretical concepts of competency like recording blood pressure (BP) were discussed in small groups. The students were encouraged to recall through drawing, flow charts, and well-labeled diagrams representing the relevant content. In the last session of the FC, students were encouraged to demonstrate the process of recording BP in a patient through role play. All the participants showed enthusiasm for peer-based and customized learning and cooperated well throughout the study. To ensure that all the students have contemplated online resources provided in the WhatsApp group, they were made to watch the videos and briefly study the PPTs and PDFs minutes before the beginning of each session (Figure 1).

After one week of completion of the FC session, a post-test was conducted with the same MCQs. There was no negative marking. A structured feedback questionnaire framed and validated by an extensive literature review was given to the students along with the post-test to understand their perception of the FC TL experience. Students were assured of confidentiality in exchange for providing an unbiased opinion.

Statistical analysis

The quantitative data were presented as mean, percentage, standard deviation, and standard error, and a paired t-test was done to understand the statistical significance of the results. The Likert scale was used to interpret the qualitative feedback. Data analysis was performed using IBM SPSS Statistics for Windows, Version 24 (Released 2016; IBM Corp., Armonk, New York, United States).

## Results

The mean scores of the post-test (19.40±4.22) were greater than those of the pre-test (12.43±4.26) confirming the significance (p-value< 0.05) of the FC (Table 1).

**Table 1:** Comparison of pre-test and post-test scores *: statistically significant, SD: standard deviation, SE: standard error, p: probability

Test	Mean	SD	SE	p-value
Pre-test	12.43	4.28	0.42	<0.05*
Post-test	19.40	4.22	0.42	

Regarding the perception of the FC, 68% (68/100) of students agreed that the objectives, methodology, and outcomes were well-defined. Around 39% (39/100) of students strongly agreed that the study material was adequate, relevant, and easy to learn. Nearly 58% (58/100) of the students agreed that the competencies are dealt with completely in every session. Roughly 16% (16/100) of students agreed that the classroom time is sufficiently utilized for peer-based learning. Many (61%, 61/100) students agreed that sufficient time was given for learning. About 34% (34/100) of the students strongly agreed that the assessment tools were relevant. About 56% (56/100) of students agreed and 21% (21/100) strongly agreed that the FC method helped them to improve their understanding of the subject. More than half (54%, 54/100) of the students agreed and 18% (18/100) strongly agreed that the FC promoted SDL. Most students (71%, 71/100) believed FC to be an interesting and satisfactory learning experience (Table 2).

**Table 2:** Student's perception of the flipped classroom MCQs: multiple choice questions

Student’s perception of the flipped classroom based on the Likert scale	Strongly disagree n (%)	Disagree n (%)	Neutral n (%)	Agree n (%)	Strongly agree n (%)
The objectives, methodology, and outcomes of the study were well-defined	00 (00)	00 (00)	26 (26)	68 (68)	06 (06)
The study material provided was adequate, relevant, and easy to learn	00 (00)	03 (03)	35 (35)	39 (39)	23 (23)
The competencies were dealt with completely in every session	00 (00)	00 (00)	22 (22)	58 (58)	20 (20)
The classroom time was utilized well for peer-based learning	00 (00)	08 (08)	34 (34)	42 (42)	16 (16)
Sufficient time was given for learning in between the sessions	00 (00)	00 (00)	12 (12)	61 (61)	27 (27)
The assessment tools like MCQs were relevant	00 (00)	03 (03)	24 (24)	39 (39)	34 (34)
The flipped classroom method helped to improve the performance in the post-test	00 (00)	00 (00)	23 (23)	56 (56)	21 (21)
The study promoted self-directed learning	00 (00)	02 (02)	26 (26)	54 (54)	18 (18)
The interaction with the mentor was beneficial	00 (00)	05 (05)	30 (30)	39 (39)	26 (26)
Overall learning experience was satisfactory	00 (00)	00 (00)	07 (07)	71 (71)	22 (22)

## Discussion

**Figure 2:**
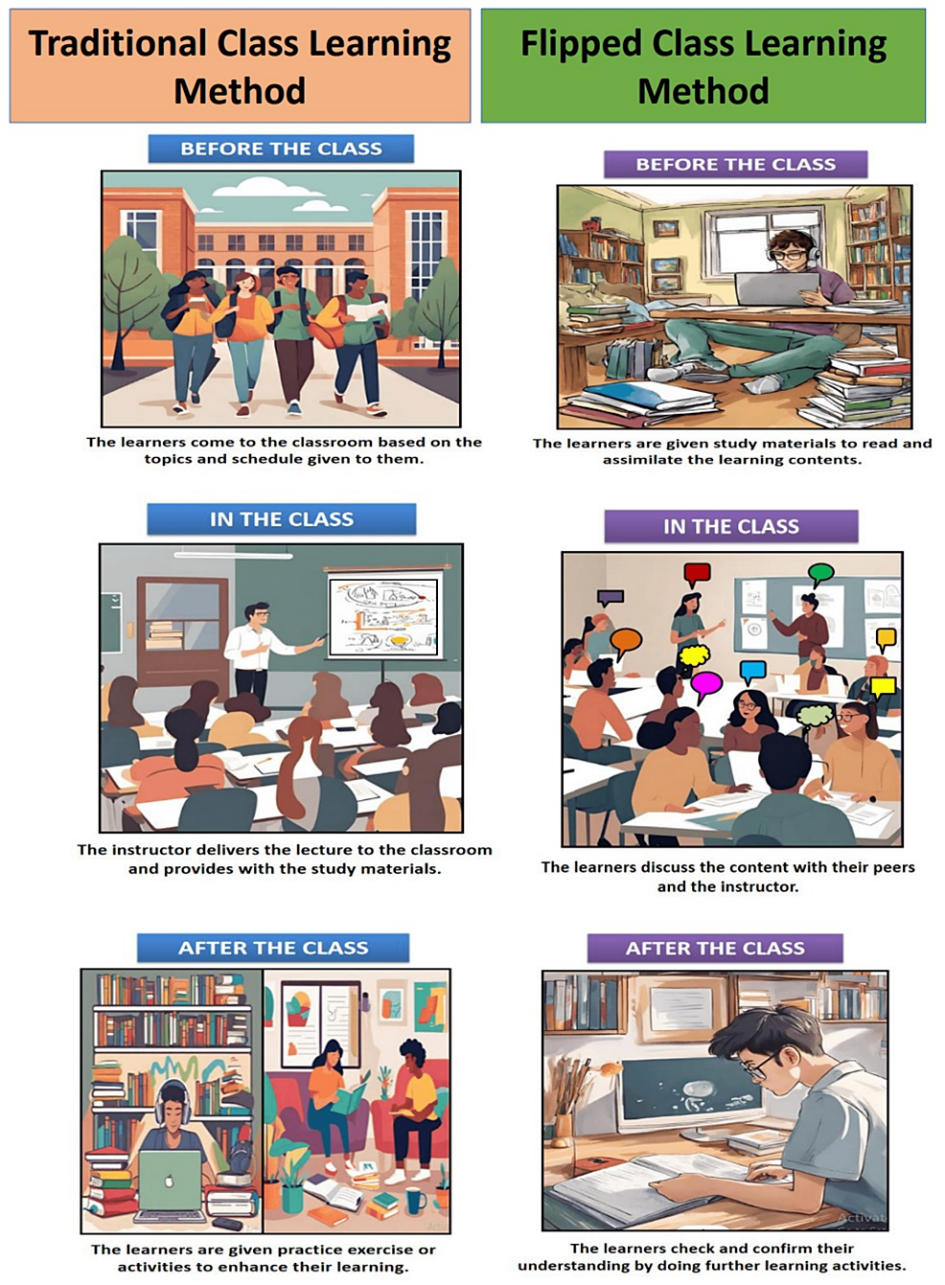
Comparison between a traditional classroom and a flipped classroom Image credit: The authors

Students pursuing MBBS are required to acquire theoretical knowledge and practice it on patients. Therefore, the TL methodology applied to impart MBBS education must be more radical and student-centered. This allows them to acquire academic knowledge and essential practical skills. In the traditional classroom, the learners are not aware of the topic of the lecture before its commencement. During lectures, the teacher delivers the knowledge and provides the learning material. After the classroom lecture, the students are provided with practical exercises. In the FC method, the students are given the relevant study material before a classroom lecture. During the classroom, the learners participate in group discussions involving peers and teachers. After the classroom, the students are encouraged to do SDL to improve their understanding of the concepts (Figure 2).

An FC centers around basic themes involving flexible environments including a shift from a routine learning culture [9]. According to Ebbinghaus’ forgetting curve, there is a direct correlation between memory and time [10,11]. The FC promotes short-term and long-term retention of conceptual and factual content. The FC is a perfect tool to motivate students to utilize the pre-class time to contemplate a large volume of relevant educational information [12,13]. Additionally, the FC allows students to collaborate during the in-class sessions and promotes student-to-student sharing of knowledge and information establishing a student-teacher-learner connection [14]. The FC model enables the teacher to think about what is best for all the students in the classroom and to facilitate each student in class every day targeting the students with inferior academic performance. The FC also caters to students with different learning paces, styles, and abilities. This method provides a customizable, flexible, and stress-free TL environment, boosting students' confidence to learn subjects.

The present study results showed a statistically significant difference in the pre-test and the post-test scores. A similar study conducted by Tune et al. used the FC approach to improve academic performance (p≤ 0.05) in learning cardiovascular, pulmonary, and renal physiology when compared with traditional instruction [15]. The FC proved to be the most effective TL method that promoted critical thinking and SDL skills among the students. A study from Visakhapatnam, India, which included medical students revealed that group dynamics play a major role in helping the students to understand the subject, take feedback, reflect, and relearn the difficult concepts [16]. A study similar to the present one included low achievers and engaged them in active learning strategies to improve their learning experience and enhance academic performance [17]. The FC helps students prepare in-depth and score better in MCQ-based assessments than through traditional didactic lectures [18].

Engaging every student in all the FC sessions in team-based activities innovatively created a lot of interest and motivation to learn and relearn the core competencies. Several innovative techniques were incorporated to promote student engagement during all the in-class FC sessions. The activities include playing a team-based game of rearranging the shuffled cards with the images of various phases of the cardiac cycle in a sequence, conducting a quiz to enhance the memory to recall the concepts and to promote critical thinking and problem-solving skills, recalling rangoli to draw the flow charts and well-labeled diagrams, doing a role play on how to record BP in a patient, etc. Students showed a lot of enthusiasm to participate in all the in-class activities and encouraged each other to learn in a stress-free learning environment.

Observations from a previous study indicated that while using the FC technique, appropriate utilization of the in-class time gave the instructors significantly more opportunities to emphasize the important concepts and engage the students in problem-solving exercises similar to peer-based learning. Students could ask the faculty and peers questions during small-group discussions and team-based activities. This promotes student-teacher interaction in the classroom [19]. Having been provided with recorded video lectures and study material in the WhatsApp group, students were encouraged to review the concepts before and after the class [15].

Pre-class activities including E-learning through open-source animated video lectures, animated medical videos, and online study material helped the students understand the concepts and made learning very interesting. However, no significant improvements in the post-test score were noticed with the FC compared with traditional instruction methods [20-22].

The FC was recommended as the most appropriate technique to shift from direct learning out of the large group learning space to moving into the individual learning space, with the help of several innovative technologies [23]. It was previously suggested that incorporating the FC method in the curriculum helps medical students to attain all six levels of Bloom's taxonomy i.e. remembering, understanding, applying, analyzing, evaluating, and creating goals. Additionally, it was proved that the FC method enables the students to reorganize and recall the information, understand the basic concepts, interpret facts, summarize what has been learned, apply knowledge to actual situations, and construct creative thinking from the knowledge gained [24].

Previous research revealed that students believed the FC method motivated them to take responsibility and accountability. This allowed them to take control over their learning in terms of the pace of the study, mastery of the content, preparedness for the in-class activities, and encouraging SDL [25]. In the FC method, the role of the teacher as a facilitator and mentor can provide instant feedback to every student that gives them an insight into their strengths and weaknesses and helps them to determine the gaps in their knowledge. This encourages students to experience reflection, relearning, and improve critical thinking skills after the FC sessions [26]. The FC method helps students reduce the time invested in preparation for the end-of-course examinations and relieve stress and burnout [27]. 

In the present study, most students appreciated the FC and preferred it over traditional learning. A high level of satisfaction was found among the students due to their participation in small group-based team activities and face-to-face interaction with the teacher and fellow students. Through the FC, the learning process has been perceived as interesting wherein students share mnemonics, flowcharts, and mind maps that contribute to better understanding and recall. The unrestricted access to review the study material and the pre-recorded videos provided to them in their WhatsApp group helped them to learn at their own pace and time.

Study limitations

This study was carried out among underachievers for a short duration. It included a limited number of core competencies in physiology. The study did not include a control group and compared the effectiveness of the FC over the traditional method. The study did not carry out the theoretical assessment and practical appraisal including case scenarios and problem-solving that could have enabled appraisal of participants' critical skills and higher-order thinking abilities. The study failed to examine the long-term retention of the concepts.

## Conclusions

The results show that most students experienced the FC as an effective and innovative TL technique to comprehend core competencies. The FC method could help underachievers improve their cognitive skills, and analytical thinking and significantly enhance their examination scores. The study has demonstrated that the FC method promotes SDL and peer-based learning in the era of CBME. The results of this study have ascertained that incorporating innovative TL techniques like FC improved the understanding of difficult topics. The FC may be extremely beneficial when used among the underachievers as evidenced from the results of the present study. The FC has been found to enhance the academic performance of the students. A blended TL method involving traditional TL methodologies and modern and advanced techniques to deliver the curriculum is suggested. 
